# Expression and Variations in *EPO* Associated with Oxygen Metabolism in Tibetan Sheep

**DOI:** 10.3390/ani14040535

**Published:** 2024-02-06

**Authors:** Yue Ren, Qiming Xi, Zhaohua He, Hongxian Sun, Shaobin Li

**Affiliations:** 1 Institute of Livestock Research, Tibet Academy of Agricultural and Animal Husbandry Sciences, Lhasa 850000, China; 2 Key Laboratory of Animal Genetics and Breeding on Tibetan Plateau, Ministry of Agriculture and Rural Affairs, Lhasa 850000, China; 3 College of Animal Science and Technology, Gansu Agricultural University, Lanzhou 730070, China; xqm5217463@163.com (Q.X.); hezh@st.gsau.edu.cn (Z.H.); sunhx@st.gsau.edu.cn (H.S.)

**Keywords:** *EPO*, Tibetan sheep, SNPs, oxygen metabolism, adaptation

## Abstract

**Simple Summary:**

The hypoxa-related gene *EPO* is a key factor in erythropoiesis. In high-altitude indigenous species, the expression of and variations in the *EPO* gene are closely related to changes of altitude (oxygen concentration). In this study, the mRNA expression levels of gene *EPO* in different tissues and organs of Hu sheep (100 m) and Tibetan sheep at different altitudes (2500 m, 3500 m, 4500 m) were detected by RT-qPCR, and Kompetitive Allele-Specific PCR (KASP) was used to study the effects of *EPO* variation on plateau hypoxia adaptation in Tibetan sheep. The results suggest that variations in the *EPO* gene were correlated with some blood gas indexes of Tibetan sheep, and the expression of the *EPO* gene was related to changes in altitude.

**Abstract:**

After a long period of adaptive evolution, Tibetan sheep have adapted to the plateau environment in terms of genetics, physiology and biochemistry, but the mechanism of hypoxia adaptation has not been fully elucidated, and the functional genes and molecular mechanisms regulating the hypoxia adaptation of Tibetan sheep need to be further studied. In this study, Tibetan sheep were selected as the research object, and the mRNA expression levels of the hypoxa-related gene *EPO* in heart, lung, kidney, liver, spleen and longissimus dorsi muscle of Hu sheep (100 m) and Tibetan sheep at different altitudes (2500 m, 3500 m, 4500 m) were assessed by RT-qPCR. The SNPs loci were detected by sequencing and Kompetitive Allele-Specific PCR (KASP) technology, then the correlation between genetic polymorphism and blood gas was analyzed. The results show that the expression of the *EPO* gene was the highest in the kidney, indicating that the expression of *EPO* gene had tissue differences. The expression levels of the *EPO* gene in the heart, lung and liver of Tibetan sheep at a 4500 m altitude were significantly higher than those in Hu sheep (*p* < 0.05), and the levels in the hearts of Tibetan sheep increased with the increase in altitude. Three mutations were identified in the EPO gene, the SNPs (g.855 A > C) in exon 1 and the SNPs (g.1985 T > G and g.2115 G > C) in exon 4, which were named EPO-SNP1, EPO-SNP2 and EPO-SNP3, respectively, and all three SNPs showed three genotypes. Correlation analysis showed that g.2115 G > C sites were significantly correlated with *p*O_2_ (*p* < 0.05), and haplotype combinations were significantly correlated with *p*O_2_ (*p* < 0.05). Thesee results suggest that the expression of the *EPO* gene is altitude-differentiated and organ-differentiated, and the *EPO* gene variants have significant effects on *p*O_2_, which may be beneficial to the adaptation of Tibetan sheep to hypoxia stress.

## 1. Introduction

Oxygen molecules are the material basis of life activity. Therefore, the perception of oxygen concentration by the organism is one of the basic functions of life activity. Hypoxia, a condition characterized by low oxygen levels, poses significant challenges to living organisms. To survive in such conditions, organisms have evolved various adaptive mechanisms. One critical protein involved in the adaptation to hypoxia is erythropoietin (EPO). The *EPO* gene is a key factor in erythropoiesis, also known as erythropoietic stimulating factor, located on chromosome 7, and it encodes the glycoprotein hormone erythropoietin [1]. *EPO* has pleiotropic actions. In addition to its role in erythropoiesis, *EPO* also exerts anti-apoptotic effects on mature RBCs and protects them from hypoxic death [2,3,4]. *EPO* acts as a key regulator in response to hypoxia-inducible factor-1 (HIF-1), a transcription factor that becomes activated under low oxygen conditions [5]. HIF-1 binds to specific hypoxia-responsive elements within the *EPO* gene promoter, leading to the increased production of *EPO*. Upon secretion into circulation, *EPO* binds to its receptor (EPOR) on hematopoietic progenitor cells, promoting their differentiation into erythrocytes (red blood cells) [6,7].

Tibetan sheep are an important livestock species adapted to living at high altitudes, and thus require efficient hypoxia adaptation mechanisms. Studies have shown that animals adapted to living at high altitudes, such as Tibetan sheep [8] and yak [9], exhibit alterations in *EPO* expression and signaling as part of their low-oxygen-adaptation strategies. The *EPO* gene is expressed in the kidneys and liver of mammals, with the primary site of expression being the kidneys [10]. *EPO* expression is induced under hypoxic conditions through the activation of HIF transcription factors. In teleosts, the expression of *EPO* promotes hypoxic adaptation [11]. Studies in animal models of hypoxia adaptation have identified genetic variants of the *EPO* gene that are associated with alterations in erythropoietic response and low oxygen tolerance. These include single-nucleotide polymorphisms (SNPs) in the promoter region of the *EPO* gene that regulate its expression under hypoxic conditions [12,13]. Adjusting blood gas is also an important aspect of mammalian adaptation to the hypoxic environment of the plateau, including the partial pressure of carbon dioxide (*p*CO_2_), partial pressure of oxygen (*p*O_2_) and oxygen saturation (sO_2_) [14]. Therefore, the aim of this research is to investigate the role of *EPO* gene expression and variations in Tibetan sheep hypoxia adaptation.

## 2. Materials and Methods

The animals involved in the tests were approved by the Animal Protection Committee of Gansu Agricultural University (Date of approval: 17 June 2020; Approval No. GAU-LC-2020-056). The test animals were used in accordance with the Guidelines for the Protection and use of Animals developed by the Ministry of Science and Technology of the People’s Republic of China (Date of issue: 22 April 2006; Approval No. 2006-398.).

### 2.1. Study Objects and Sample Collection

In total, 351 Tibetan sheep were subjected to genotyping in exon1 and exon 4 of *EPO*, of which 176 (134 females and 42 males) were used for blood gas analysis. All Tibetan sheep were in good health and around 3.5 years old. These Tibetan sheep live in a herder’s flock at an altitude of more than 2800 m in Maqu County, Gansu Province, China. A 5 mL sodium heparin collection tube was used to collect blood from the jugular vein of each sheep; a small amount of each blood sample was taken for some of the blood gas index analyses, and the rest of the blood samples were collected and preserved with TFN paper (Munktell Filter AB, Falun, Sweden) for the two-step process of extracting DNA [15].

For *EPO* gene expression analysis, nine Tibetan sheep were randomly selected, among which three were distributed in Zhuoni County, Gansu Province, China (2500 m), three were distributed in Haiyan County, Qinghai Province, China (3500 m), three were distributed in Zhiduo County, Qinghai Province, China (4500 m), and three Hu sheep were obtained from Kangrui Breeding Sheep Co., Ltd. in Baiyin City, Gansu Province, China (1800 m). All sheep were in good health and around 3.5 years old (ewes). Because rams of the same age in the experimental sample were breeding rams, only ewes were selected for slaughter. Sodium pentobarbital (350 mg) was injected intravenously into each experimental sheep, and dissection was performed after the heart stopped beating and continuous involuntary respiration had ceased for about 3 min. Then, the heart, liver, spleen, lungs, kidneys and longissimus dorsi muscle were collected for RT-qPCR. The collected tissue samples were immediately placed in liquid nitrogen and transferred to the laboratory, and then stored at −80 °C to extract the total RNA.

### 2.2. Blood Gas Indicator Measurement

Blood gas physiological indices such as partial pressure of oxygen (*p*O_2_), partial pressure of carbon dioxide (*p*CO_2_), pH, and oxygen saturation (sO_2_) were directly measured using an i-STAT blood gas analyzer (Abbott, Chicago, IL, USA). In addition, the partial pressure of oxygen at which hemoglobin is 50% saturated with oxygen (*p*_50_) was calculated using *p*O_2_, sO_2_, and pH [16]. The following equations were used.

$$p_{50}\;\mathrm{std}=anti\;\log\frac{\log\left(\frac{1}{k}\right)}{n};\;where\;\frac{1}{k}=\left[anti\log(n\log pO_{2\left(7.4\right)})\right]\cdot \frac{100-{\mathrm{sO}}_{2}}{{\mathrm{sO}}_{2}}$$

A Hill constant “*n*” for hemoglobin A of 2.7 was used. The (*p*O_2_ in venous blood at 37 °C was converted to *p*O_2_ at pH = 7.4 using the following equation:

$$\log pO_{2\left(7.4\right)}=\log pO_{2}-\left[0.5\;\left(7.40-\mathrm{pH}\right)\right]$$

To ensure the validity of parametric tests, the normality assumptions of these data were examined using Kolmogorov–Smirnov tests by SPSS.

### 2.3. Primers for PCR and RT-qPCR

According to the gene sequence of sheep *EPO* (entry number: NM_001024737.1) published by GenBank, primers were designed for exon 1 and exon 4 of the *EPO* gene. The primers were designed using the Premier 5.0 software (Table 1). All primers were synthesized by Wuhan Okodingsheng Biotechnology Co., Ltd. (Wuhan, China). *β-actin* (accession number: NM_001009784) was used as the reference gene.

RT-qPCR was performed by the relative quantitative SYBR Green 1 dye method. The reaction system was 20 μL in total: 7.2 μL RNase-Free water, 10 μL 2 × Premix, 0.8 μL (10 ng/μL) upstream and downstream primers, and 2 μL cDNA (100 ng/μL). Reaction conditions: predenaturation at 95 °C for 5 min, denaturation at 94 °C for 15 s, annealing for 30 s (refer to Table 1 for annealing temperature), 40 cycles, and then dissolution curve analysis. 2^−ΔΔCt^ [17] was used to analyze the relative expression levels of the *EPO* gene. Three biological replicates and four technical replicates were performed to ensure that the findings were genuine and trustworthy.

### 2.4. Genotyping

Firstly, the blood DNA from 20 Tibetan sheep was used and sequenced after the amplification of exon 1 and 4 fragments. Primer synthesis, fragment amplification and sequencing were performed at Shanghai Sangon Biotech Co., Ltd. (Shanghai, China). After the discovery of SNPs by sequencing, each SNP was genotyped by KASP technology applied to the 351 Tibetan sheep samples, and then the genotyping map was generated using LGC-OMEGA 5.0 software, which was done at Wuhan Gentides Biotech Co., Ltd. (Wuhan, China).

### 2.5. Statistical Analyses

The original experimental data in this study were sorted and statistically analyzed using Excel 2010 software, and significance analysis was performed using SPSS 26.0 software. All bar charts were produced using the Prism 9.0 software. One-way analysis of variance (ANOVA) was used to compare the mean values among different genotypes. The least significant difference (LSD) post hoc test was performed for multiple comparison. The data were expressed as mean ± standard error, and *p* < 0.05 was considered statistically significant.

After successful *EPO* genotyping using the KASP assay, allele frequency, genotype frequency, genetic homozygosity (Ho), genetic heterozygosity (He), effective allele number (Ne) and polymorphism information content (PIC) were calculated using the formulas described by Botstein et al. [18]. A Chi-square test was performed to detect the Hardy–Weinberg equilibrium; *p* > 0.05 indicated that the population met the Hardy–Weinberg equilibrium, otherwise it did not meet the Hardy–Weinberg equilibrium. The SNPs linkage disequilibrium and haplotype of *EPO* gene were analyzed using Haploview4.1 and PHASE 2.0 software.

## 3. Results

### 3.1. Tissue Expression Characteristics of EPO Gene

**Figure 1:**
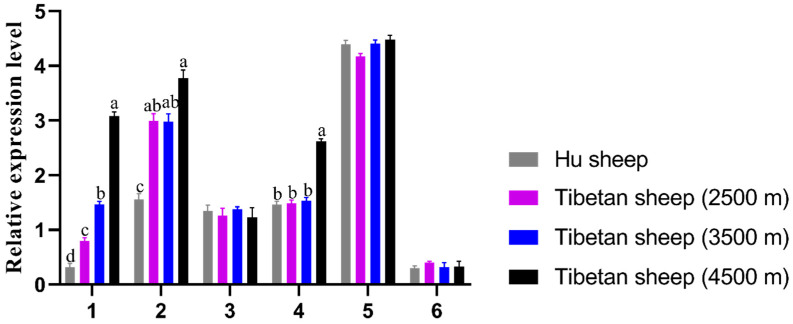
Relative expression levels of *EPO* mRNA in different tissues of Hu sheep and Tibetan sheep from different altitudes. Note: The abscissa numbers 1, 2, 3, 4, 5 and 6 represent the heart, liver, spleen, lungs, kidney and longissimus dorsi tissue, respectively. In the legend, “Hu sheep” represents the Hu sheep, “Tibetan sheep (2500 m)” represents the Tibetan sheep at 2500 m altitude, “Tibetan sheep (3500 m)” represents the Tibetan sheep at 3500 m altitude, and “Tibetan sheep (4500 m)” represents the Tibetan sheep at 4500 m altitude. The bars represent the mean ± SE from three independent biological replicates, each performed with four technical replicates. The different letters indicate significant differences (*p* < 0.05), the same letter indicates no significant differences (*p* > 0.05).

The mRNA expressions of the *EPO* gene in different tissues of Tibetan sheep and Hu sheep are shown (Figure 1). The *EPO* gene was expressed in six tissues of Hu sheep and Tibetan sheep at different altitudes to varying degrees, and the expression of the *EPO* gene was the highest in the kidneys of the two breeds of sheep, while the expression was the lowest in the muscle of the two breeds of sheep, indicating that the expression of this gene had tissue differences. In the heart and lung, the expression of this gene in Tibetan sheep at 4500 m altitude was significantly higher than that in Tibetan sheep at other altitudes and Hu sheep (*p* < 0.05), and the expression of this gene tended to increase with the decrease in oxygen concentration caused by the increase in altitude. In the liver, the expression of this gene was significantly higher in Tibetan sheep at all three altitudes than in Hu sheep (*p* < 0.05).

### 3.2. Variation of EPO in Tibetan Sheep

**Figure 2:**
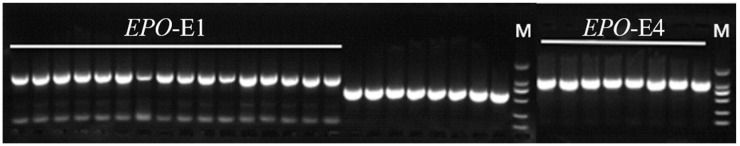
Electrophoresis of *EPO* gene PCR amplification products of Tibetan sheep. The lanes labeled EPO-E1 and EPO-E4 are amplified products of exon 1 and exon4 of *EPO* gene, respectively. The lanes labeled M are the DNA marker.

**Figure 3:**
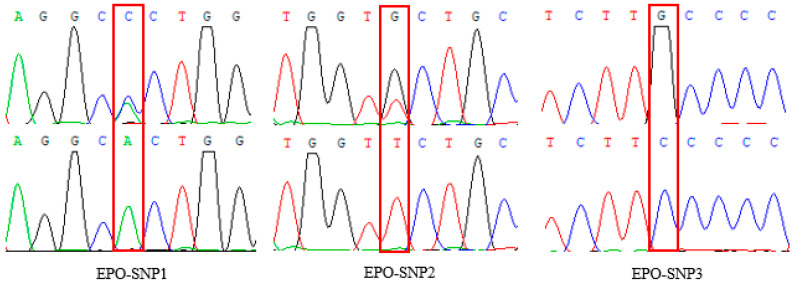
Sequencing results of *EPO* SNPs’ PCR amplification products. The sites in the box are SNP sites.

**Figure 4:**
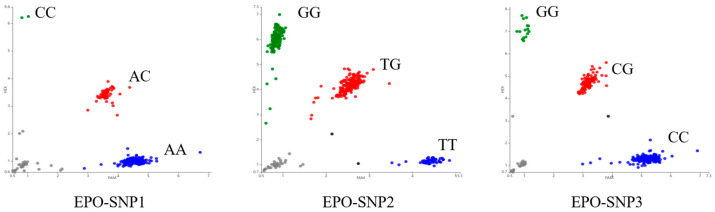
KASP genotyping assay results of three loci of the *EPO* gene in Tibetan sheep. Horizontal and vertical coordinates for the two joints recognized by mutant base signaling, respectively. FAM is blue, HEX is green, and FAMHEX is red. Blue and green dots represent homozygotes and red dots represent heterozygotes. Gray dots represent unrecognized signals.

The amplification products of the *EPO* gene were detected by 1.5% agarose gel electrophoresis. The bands of the amplification products were complete, clear, and non-specific; their sizes were in line with the expected results, and the amplification products could be directly used for subsequent PCR product sequencing (Figure 2). The *EPO* gene sequences of 20 randomly selected Tibetan sheep were sequenced. After sequence comparison, one SNP locus, g.855 A > C, was detected in exon 1 of *EPO* gene, and two SNPs loci, g.1985 T > G and g.2115 G > C, were detected in exon 4 of the *EPO* gene. Among them, g.2115 G > C is a missense mutation, where glycine changes to alanine, and the other two sites are synonymous mutations. The three loci were named g.855 A > C, g.1985 T > G and g.2115 G > C, respectively (Figure 3). The three SNPs were genotyped by the KASP assay, and all three genotypes were present in Tibetan sheep: *AA*, *CC*, *AC* (g.855 A > C)/*GG*, *TT*, *GT* (g.1985 T > G)/*CC*, *GG*, *CG* (g.2115 G > C), respectively (Figure 4).

### 3.3. Population Genetic Diversity Analysis of EPO Gene

The *AA* genotype frequency of g.855 A > C was the highest (0.8195), and the *A* allele was the most frequent allele. The frequency of *GT* genotype at the g.1985 T > G locus was the highest (0.5029), and the *G* allele was the most frequent allele. The frequency of *CC* genotype at g.2115 G > C was the highest (0.6905), and the *C* allele was the most frequent allele (Table 2). The three SNPs of *EPO* gene were consistent with Hardy–Weinberg equilibrium in the Tibetan sheep population (*p* > 0.05), while the homozygosity (Ho) was high and the heterozygosity (He) was low. The number of effective alleles of g.1985 T > G (1.9077) was the highest, and g.855 A > C (1.2031) was the lowest. g.855 A > C and g.2115 G > C were in low polymorphism (PIC < 0.25) and g.1985 T > G was in moderate polymorphism (0.25 < PIC < 0.5) (Table 3).

### 3.4. Effect of Variation in EPO on Blood Gas

The g.2115 G > C site was significantly correlated with *p*O_2_ (*p* < 0.05), and the *p*O*_2_* of *TT*-type individuals was significantly higher than that of *GG-* and *GT*-type individuals (*p* < 0.05), but there was no significant difference between *GG-* and *GT*-type individuals (*p* > 0.05). (Table 4) The results indicate that the data for these four traits all follow a normal distribution, with *p* values for *p*O_2_, *p*CO_2_, sO_2_ and *p*50 of 0.098, 0.067, 0.052 and 0.203, respectively.

### 3.5. Analysis Linkage Disequilibrium

**Figure 5:**
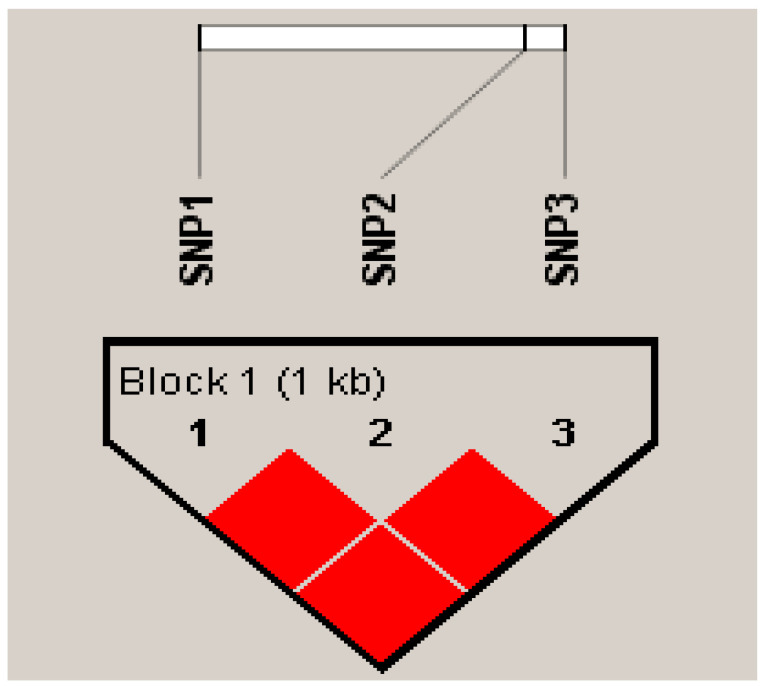
SNP linkage disequilibrium analysis of the *EPO* gene. These were in a fully linked state (R^2^ = 1).

The linkage disequilibrium and haplotype of SNPs in the Tibetan sheep *EPO* gene were analyzed using Haploview 4.2 software. A haplotype block can be constructed at the three SNPs sites of the *EPO* gene, all of which are in a fully linked state (R^2^ = 1) (Figure 5). After the construction of the *EPO* gene haplotypes, there were four haplotypes with frequencies greater than 0.05, namely, H1 (ACT, 0.390), H2 (ACG, 0.344), H3 (AGG, 0.171) and H4 (CCG, 0.096). By combining the four haplotypes of the *EPO* gene, a total of 7 combinations with frequencies greater than 0.03 were obtained (Table 5).

### 3.6. Correlation Analysis between Haplotype Combination and Blood Gas

Correlation analysis was conducted between seven haplotype combinations of *EPO* gene and blood gas indexes of Tibetan sheep. Seven different haplotype combinations of the *EPO* gene were significantly correlated with *p*O_2_ (*p* < 0.05), among which the *p*O_2_ of H2H4 was significantly higher than that of H2H2 and H2H3, and the seven haplotype combinations had no significant association with other blood gas indexes (*p* > 0.05) (Table 6).

## 4. Discussion

Oxygen, like water and food, is necessary for the life of mammals. For aerobic animals, a change of oxygen concentration can cause a stress response in the body, and hypoxia will lead to the damage of tissue and organ function, and then affect the normal physiological function of the body [19]. Hypoxic adaptation, also known as hypoxic response, refers to an adaptive self-protection mechanism initiated by the body to maintain normal physiological functions under hypoxic stress [20]. To cope with this pressure, high-altitude indigenous animals have evolved unique adaptive strategies for circulation, respiration, and blood tolerance, including single-nucleotide variation, copy number variation, differential gene expression, isoforms, transposable elements, and methylation [21]. The core of the hypoxic response mechanism is hypoxic-inducing factor HIFs, which is composed of two subunits, α and β, in which α is a functional subunit (including 1α, 2α and 3α) and β is a structural subunit [22,23]. Hypoxia-inducible factor (HIFs) is a response regulator that mediates the adaptive response of cells under hypoxia conditions, and regulates the expression of hypoxia-related genes. Erythropoietin (EPO), vascular endothelial growth factor (VEGF) and platelet-derived growth factor (PDGF) are the downstream genes of HIFs, and their expression is regulated by HIFs [24].

Under hypoxic conditions, the *EPO* gene is widely expressed, plays an important role in erythropoiesis and tissue protection, and participates in many physiological processes [25,26]. *EPO* expression is the highest during hypoxia, when the body needs to increase red blood cell production to compensate for the reduced oxygen levels. Studies have shown that the expression of *EPO* in the kidney and liver of Tibetan sheep is higher than that of plain sheep [25], and it was also found that *EPO* levels rise in human plasma with increasing altitude [27]. The present study also found that the expression of *EPO* gene in the liver of Tibetan sheep was higher than that of Hu sheep, suggesting that *EPO* concentrations may be related to altitude (oxygen levels). Additionally, we observed the tissue-specific expression of *EPO*, with particularly high expression levels in the kidney, suggestive of their potential involvement in regulating hematopoiesis and hypoxia adaptation. In this study, it was found that the expression of *EPO* in the heart showed an increasing trend with the increase in altitude. The heart is an oxygen-demanding organ that relies on a continuous supply of oxygen to produce mechanically functional high-energy phosphate ATP, and the high expression of *EPO* in this organ with increasing altitude in sheep confirms the association of this gene with oxygen metabolism. These results suggest a potential role for this gene in determining the adaptability and fitness of Tibetan sheep in high-altitude environments.

In the plateau environment, due to the lower oxygen concentration, the animal organism requires a larger amount of red blood cells to provide sufficient oxygen. At this point, the variation in the *EPO* gene is important for animals to adapt to the plateau environment [28]. However, the specific genetic variants that contribute to this adaption are not fully understood. SNPs are the most common type of heritable variation and are widely present in the genome. Because SNPs are associated with many production traits of economic animals, they are thus the focus of methods to improve production performance. Therefore, SNPs are increasingly being used in research as a molecular genetic marker method [29]. In this study, three SNPs were detected in the *EPO* gene sequence, and population genotyping showed that the three SNPs of the *EPO* gene had three genotypes, among which the *p*O_2_ of *CC* and *CG* individuals at the g.2115 G > C locus was significantly lower than that of *GG* individuals (*p* < 0.05). There was no significant difference in *p*O_2_ between *CC*-type and *CG*-type individuals. Studies have shown that the body adaptively increases lung ventilation, heart rate, and total blood volume in order to ensure that there is enough oxygen to bind to hemoglobins in hypoxic environments. In tissues with low *p*O_2_ levels, oxygen often dissociates; in other words, low *p*O_2_ facilitates the separation of oxygen from hemoglobin [30], and the three genotypes had different hemoglobin-binding abilities to oxygen, which also suggests that the difference in blood gas index among different genotypes is mainly caused by the difference in altitude. Therefore, compared with *GG*-type individuals, *CC*- and *CG*-genotype individuals may have a stronger oxygen carrying capacity, and better adapt to the plateau environment. Haplotype analysis showed that the H2H2 and H2H3 haplotype combination had a lower *p*O_2_, and this made up 17% of the population, which suggests that the adaptability of these sheep may differ from that of other sheep. 

Despite the progress made in understanding the role of *EPO* in hypoxia adaptation, efforts should be made to identify novel genetic variants of the *EPO* gene associated with hypoxia tolerance and production performance in plateau animals. Such knowledge will not only enhance our understanding of hypoxia adaptation, but also contribute to the development of more efficient breeding strategies for Tibetan sheep and yak, and other plateau livestock. But this requires more in-depth research in different sheep breeds and other species.

## 5. Conclusions

The expression of the *EPO* gene was closely related to changes in altitude (oxygen concentration), and the expression of *EPO* in some organs showed organ-specific and varietal differences. The variation in the *EPO* gene was correlated with some blood gas indexes of Tibetan sheep; that is, the g.2115 G > C site was significantly correlated with *p*O_2_ (*p* < 0.05). Seven different haplotype combinations of the *EPO* gene were significantly correlated with *p*O_2_ (*p* < 0.05), and the *p*O_2_ of H6H8 was significantly higher than those of H6H6 and H6H7.

## Figures and Tables

**Table 1:** Primers for PCR and RT-qPCR.

Gene	Exon	Primer Sequence (5′→3′)	Product Size (bp)	Annealing Temperature/°C	Purpose
*EPO*	1	F: TTGAAGTTTGGCCGGGAGAAGTG R: CCAAGCCAGCCTCACTCTCCTCC	864	58	PCR
*EPO*	4	F: GCTGTTTCTGTAAAGTGAGAGAAGGGC R: GCCAGTGGGGTCAAGAGGTCAG	1008	57	PCR
*EPO*		F: CTGTCTTTTCTGCTGTTTCCTCTG R: CTCCATCCTCTTCCAGGCATA	207	60	RT-qPCR
*β-actin*		F: AGCCTTCCTTCCTGGGCATGGA R: GGACAGCACCGTGTTGGCGTAGA	113	60	RT-qPCR

**Table 2:** Genotype frequency and gene frequency of three SNPs’ loci in *EPO* gene.

SNP Site	Number	Genotype Frequency (Number)			Allele Frequency	
g.855 A > C	349	*AA*	*AC*	*CC*	A	C
		0.8195 (280)	0.1748 (61)	0.0057 (2)	0.9069	0.0931
g.1985 T > G	346	*GG*	*GT*	*TT*	G	T
		0.3584 (124)	0.5029 (174)	0.1387 (48)	0.6098	0.3902
g.2115 G > C	349	*CC*	*CG*	*GG*	C	G
		0.6905 (241)	0.2665 (93)	0.0430 (15)	0.8238	0.1762

**Table 3:** The population genetic diversity of three SNPs of the *EPO* gene.

SNP Site	Ho	He	Ne	PIC	Hardy–Weinberg *p*-Value
g.855 A > C	0.8312	0.1688	1.2031	0.1545	*p* > 0.05
g.1985 T > G	0.5242	0.4758	1.9077	0.3625	*p* > 0.05
g.2115 G > C	0.7096	0.2904	1.4092	0.2483	*p* > 0.05

**Table 4:** The effects of genotypes of *EPO* SNPs on blood gas.

SNP Site	Blood Gas	Genotype		
g.855 A > C		*AA* (*n* = 286)	*AC* (*n* = 61)	*CC* (*n* = 2)
	*p*CO_2_	40.453 ± 0.643	41.742 ± 0.464	40.557 ± 0.372
	*p*O_2_	38.618 ± 0.365	38.364 ± 0.546	38.037 ± 0.407
	sO_2_	70.587 ± 0.823	70.561 ± 0.554	71.701 ± 0.865
	*p*_50_	26.470 ± 0.783	26.669 ± 0.059	26.256 ± 0.697
g.1985 T > G		*GG* (*n* = 124)	*GT* (*n* = 172)	*TT* (*n* = 48)
	*p*CO_2_	40.236 ± 0.431	41.057 ± 0.758	40.531 ± 0.478
	*p*O_2_	39.465 ± 0.668	38.380 ± 1.445	38.624 ± 0.930
	sO_2_	70.493 ± 0.292	70.562 ± 0.281	70.537 ± 0.840
	*p*_50_	26.764 ± 0.842	27.201 ± 0.329	26.684 ± 0.506
g.2115 G > C		*CC* (*n* = 241)	*CG* (*n* = 93)	*GG* (*n* = 15)
	*p*CO_2_	40.127 ± 0.473	40.757 ± 0.437	40.140 ± 0.356
	*p*O_2_	38.365 ± 0.566 ^b^	38.064 ± 0.442 ^b^	43.404 ± 0.494 ^a^
	sO_2_	70.642 ± 1.537	70.066 ± 0.457	70.791 ± 1.209
	*p*_50_	27.537 ± 0.802	26.729 ± 0.593	26.371 ± 0.401

Note: Estimates are given as X ± SE; data in the same line are significantly different with different letters (*p* < 0.05), and not significantly different with the same letters (*p* > 0.05). *n*: The number of observed sheep. Same as below.

**Table 5:** Frequency of haplotype and haplotype combination of SNP locus reconfiguration in the *EPO* gene.

Haplotype	SNP1	SNP2	SNP3	Frequency/%	Haplotype Combination	Haplotype Combination Frequency/%
H1(ACT)	A	C	T	0.390	H1H1	0.1521
H2(ACG)	A	C	G	0.344	H1H2	0.1342
H3(AGG)	A	G	G	0.171	H1H3	0.0667
H4(CCG)	C	C	G	0.096	H1H4	0.0374
					H2H2	0.1183
					H2H3	0.0588
					H2H4	0.0330

**Table 6:** Effects of *EPO* gene reconfiguration haplotype on blood gas.

Haplotype Combination	*p*CO_2_	*p*O_2_	sO_2_	*p*_50_
H1H1	40.347 ± 0.640	38.725 ± 0.305 ^ab^	70.621 ± 0.773	26.484 ± 0.333
H1H2	40.821 ± 1.063	37.923 ± 0.127 ^ab^	70.081 ± 0.493	26.880 ± 0.453
H1H3	41.776 ± 0.902	38.276 ± 0.884 ^ab^	69.796 ± 0.545	26.633 ± 0.892
H1H4	40.901 ± 0.258	38.190 ± 1.896 ^ab^	70.278 ± 0.984	26.043 ± 1.574
H2H2	41.670 ± 0.515	36.551 ± 0.295 ^b^	70.683 ± 0.998	26.499 ± 0.339
H2H3	39.754 ± 0.862	37.629 ± 0.653 ^b^	69.850 ± 0.739	25.971 ± 0.509
H2H4	40.573 ± 0.163	40.304 ± 0.796 ^a^	70.801 ± 1.473	26.302 ± 0.331

Note: Different letters indicate significant differences (*p* < 0.05).

## Data Availability

The raw data supporting the conclusions of this article will be made available by the authors on request.
